# Consensual qualitative research on the internship experience and development of career identity of Korean doctors

**DOI:** 10.1186/s12909-020-02451-4

**Published:** 2021-01-06

**Authors:** Hye-Jin Lee, Moonsang Ahn

**Affiliations:** 1 Chungnam National University Hospital, Planning & Budget Division, Daejeon and Department of Human Resource Development, Korea University of Technology and Education, Cheonan, South Korea; 2 Department of Surgery, Chungnam National University College of Medicine, 282 Munhwa-ro, Jung-gu, Daejeon, 301-721 South Korea

**Keywords:** Intern, Resident, Specialty, Qualitative research, Career identity

## Abstract

**Background:**

Career identity is defined as the ability to substantialise career goals and results from the social learning process achieved through interactions with others.

This study aimed to understand how the internship experience in Korea affects career identity, which provides a foundation for developing professional values while promoting personal goals and aspirations.

**Methods:**

We conducted eleven semi-structured interviews with interns at a university hospital in Korea who had completed internships and chosen a speciality. The interview transcripts underwent inductive thematic analysis using consensual qualitative research approaches. Themes identified were categorised from three domains for the year-long internship experience: personal cognitive, social interaction, and system domains.

**Results:**

Researchers derived seven categories and 20 subcategories from the transcripts: (1) self-reflection throughout internship training, (2) practical awareness of the internship programme’s operation, (3) perception of individual competence, (4) recognition of mentor importance, (5) situational awareness in the clinical department, (6) relationship experience, and (7) experience of institutional limitations. The internship experience, during which the individual is in charge and core values drive career decisions, is important for the formation of career identity and career orientation.

The internship programme provides information about the clinical department to applicants seeking residency and serves a mediating role, providing information about applicants to the clinical departments. Internship is an important period during which career identity is formed.

**Conclusion:**

The internship programme provides information about clinical departments to applicants seeking residency; it is an important period during which career identity is formed. This study helps provide an in-depth understanding of interns and a base for developing institutional and policy support for students during an uncertain time when specialties should be selected.

## Background

In Korea, graduates of a college or graduate school of medicine can legally provide medical treatment immediately after graduating, upon obtaining a Korean medical licence. Although education in primary-care medicine is necessary, over 90% of doctors who obtain medical licences in Korea are specialists [1]. Four or 5 years of training is necessary to become a specialist. First year trainees are called ‘interns’ and they choose their specialty following the internship, during the residency selection process. Personal and social interactions experienced at the new clinical learning site, that is the hospital, −constitute important variables for future career choices [2] and interns explore and plan their future specialty through their experiences in this context.

If a career is seen as a ‘lifetime experience or activity’ [3], the internship period is critical for gaining career experience, and establishing career identity, prior to choosing a specialty [4, 5]. Career identity is the ability to orientate oneself and grow in a changing environment. It constitutes an understanding of oneself and of what to pursue in the future, and the ability to substantialise and achieve career goals [6]. It is also a career orientation, where the individual is in charge and where core values drive career decisions [7]. It results from a social learning process that takes place through interaction with others [5].

Meijers [8] suggested that personal cognitive activities and experiences in the professional world form new meanings that enhance career identity and prompt reflection on experiences toward valuable results. Pursuing a career is considered important in the current professional environment [9] and career identity is key to self-directed career management based on individual values. Career identity development is especially necessary in the medical field, as the specialty that may become a lifelong career is determined after the internship. Thus, it is important to understand interns’ thoughts, conflicts, and experiences throughout the process of deciding a specialty.

Related previous research includes quantitative studies and surveys on medical students, interns and residents, along with questionnaire development regarding specialty choices [10, 11], and analyses of lifestyle and trends in specialty choice [12–15]. However, these studies do not offer an in-depth explanation of the complex career identity formation process. A quantitative approach to the career identity formation process is limited as it cannot fully account for the individual experience at the centre of such processes.

Thus, this study explores how the internship process, internship experiences, and interns’ perceptions influence career identity formation, as well as the internship system’s efficiency, with a qualitative approach. Based on existing research, this study divides career identity formation into the personal cognitive and social interaction domains. The system domain was added with consideration for the Korean internship system’s special nature. The study examines career identity from each perspective and proposes future research that may help improve interns’ career identity.

Our research questions are as follows:

1. *What factors influence formation of interns’ career identity?*
2. *How does the internship process affect formation of interns’ career identity?*

## Methods

This study aims to give an in-depth account of medical residents’ experiences during the internship process. Researchers in the field of medical education and training have advocated qualitative research as the most suitable approach toward this aim [16]. In particular, such researchers have emphasised a consensual qualitative research (CQR) approach as appropriate [17]. CQR is a qualitative analysis method that combines Strauss and Corbin’s grounded theory with Elliott’s comprehensive process analysis and is a systematic method of reaching consensus through core concept extraction, subtraction, and cross analysis to overcome previous limitations to qualitative research [18]. Understanding and analysing the data through repeated consensus among multiple researchers helps the prevention of bias and meaningful omissions in the interpretation of data.

The participants of the study had completed internships at a university hospital in Korea and had decided their speciality. Selection was conducted by an intern representative who explained the research purpose to the interns and proceeded with snowball sampling. Finally, 11 interns were selected. As results of qualitative studies may vary depending on gender, the gender ratio was evenly set (6 men, 5 women).

After lengthy discussions, the final questions were determined by the researchers:

1. *Tell me about your internship experience, including your experience as a medical student.*
2. *How did you feel about your future specialty (career) when you were in the clinical department and the internship programme?*
3. *Which experience made you prefer or avoid certain specialities?*
4. *Have there been changes to your specialty choices?*
5. *If there were changes, which factors do you think influenced the changes? (Personal, social, and system domains)*

The interviews took place from December 2016 to February 2017 and took about an hour. After obtaining consent from the study participants, recordings and field notes were prepared and used as supplementary data in the analysis. After the interviews, the researchers confirmed and used data transcribed by the research assistant for the analysis.

This study’s consensus team included one doctoral student from the Department of Human Resource Development and two professors from the College of Medicine. The study’s supervisor majored in education and is currently a professor in the Department of Human Resources Development. Data analysis took place at weekly meetings over 5 months. We went through a cross tabulation analysis and editorial process that categorised the domain coding and key contents. This study was conducted after obtaining approval from the institutional review board of Chungnam National University Hospital (IRB File No. CNUH 2016–10–029-001).

## Results

Researchers derived seven categories and 20 subcategories from three domains (personal cognitive, social interaction, and system domains) for the year-long internship experience process. In Table 1, ‘Category/subcategory’ lists the meaningful topics obtained from the data collected in the interviews. If the extracted concept appeared in categories that corresponded to all participants or to all participants minus one, it was labelled as ‘general’. Content that appeared in over 50% of cases was ‘typical’; in fewer than 50%, ‘variable’. Interview cases for each area are presented selectively because of limited space.

**Table 1:** Personal cognitive domain

Domain	Category/Subcategory	Frequency
Personal cognitive domain	1) Self-reflection throughout internship training	
	a. Improved understanding of the clinical department	Typical (9)
	b. Reduced fear through workplace-based training	Variable (2)
	c. Self-awareness of career preferences	General (10)
	2) Practical awareness of the internship programme’s operation	
	a. Recognising the internship programme’s necessity	Variable (4)
	b. Dissatisfaction with simple labour transfer	Typical (6)
	c. Dissatisfaction with training duration and limited number of training departments	Variable (4)
	3) Perception of individual competence	
	a. Experience of the lack of general competency	Variable (3)
	b. Worry about the limitation of future professional competency	Variable (3)

### A. Personal cognitive domain

In this area, three categories related to personal cognitive domain were derived: ‘self-reflection through internship training’, ‘practical awareness of the internship programme’s operation’, and ‘perception of individual competence’ (Table 1).

#### Self-reflection throughout internship training

In this area, categories related to introspection through internship training were derived, including ‘improved understanding of clinical departments’ and ‘self-awareness of career preferences’; additionally, some participants reported reduced fear throughout internship training. Although there was significant psychological stress from monthly departmental changes, many interns believed that internship opportunities—such as participation in surgery or the intern attending-physician system—provided an in-depth experience. Through the experience, interest arose in specialties not considered before (Participant 10).

Participant 4 recalled that many interns, who became very sensitive to patients’ conditions and felt guilty when their conditions deteriorated, overcame their feelings of guilt and fear through on-site education and encouragement from senior residents.

*I was a little scared. I have to become a resident, but what if something happens to the patient because I’m stupid? That time, the residents said, ‘Even if you’re not memorising all of Harrison, we’ll educate you. There’s a routine protocol, so don’t worry too much’. And I kept working and settled down. (Participant 4)*

Internship training offers environment-specific development that provides individuals with opportunities to reflect on their careers in a self-reliant, proactive manner.

*I was a little sceptical about Rehabilitation because it isn’t much of a specialty if it’s just a brief process following the emergency stage before being discharged. But there’s a different approach that only a Rehabilitation physician has when it comes to seeing a patient, such as physical examination with a precise rehabilitative goal. (Participant 5)*

#### Practical awareness of the internship Programme’s operation

This area related to the internship programme’s necessity, dissatisfaction with simple labour transfer, dissatisfaction with training duration, and the limited number of training departments. Because of lack of clinical skill and experience, many interns initially expressed difficulties in chaotic situations involving on-the-scene medical care. They were highly aware of their limitations, recognising the importance of the internship programme for developing confidence and skills. However, some interns acknowledged that being assigned repetitive and simple tasks constituted labour transfer, criticising the limited training period and number of training departments included in the internship; 1 year was too short to experience all departments, they argued.

*As an attending physician, I was able to put together the puzzle pieces I gathered through the lectures. In one case, a patient’s blood pressure dropped drastically, and it was great to comprehensively use what I had learned theoretically with the patient, like how much urine output signifies shock, checking the amount of urine to confirm if it’s shock, and which medication for raising blood pressure. (Participant 9)*

*Frankly, finding a blood vessel and sticking a syringe to draw blood is simple. Doing that for a year didn’t make me feel worthwhile as a doctor. (Participant 5)*

*The internship is necessary, but is doing it for a year necessary? …I think an internship could be unnecessary if you could experience the department in sub-intern [an experience-based course] or some other way. (Participant 7)*

#### Perception of individual competence

In this area, we examined the professional and general competency limitations of interns who, acting as doctors, treat patients.

One participant said that the interest and effort to resolve conflicts with patients naturally led her to study customer satisfaction. The participant learned that walking out during a patient conflict worsened the situation and realised that a highly emotionally supportive personality is necessary, but she did not want such efforts to be revealed to colleagues. She further stated that although the training hospital offered various job training sessions, it was impossible to participate in them during the internship (Participant 4).

*I've heard that if I want to do a liver transplantation, I have to become a fellow like a resident and practise for 10 years. Considering my age, will my hands not tremble after 10 years? (Participant 2)*

### B. Social interaction domain

Career identity is formed through personal cognitive processes and continuous interaction with one’s environment. Meijers [8] categorised social interaction domains that affect personal cognitive domains as: ‘recognition of mentor importance’, ‘situational awareness in the clinical department’, and ‘relationship experience’ (Table 2).

**Table 2:** Social interaction domain

Domain	Category/Subcategory	Frequency
Social interaction domain	1) Recognition of mentor importance	
	a. Psychological support from senior residents	Typical (6)
	b. Positive support experience from professors	Typical (7)
	2) Situational awareness in the clinical department	
	a. Experience of the reality of the desired specialty	Typical (7)
	b. Residents’ quality of life	Typical (6)
	c. Consideration of competition	Variable (5)
	d. Experiences related to gender discrimination	Variable (2)
	3) Relationship experience	
	a. Preliminary approval of decision by the department	Variable (5)
	b. Experience of the relationship with patients	Variable (4)

#### Recognition of Mentor importance

Participants contemplating several specialties were able to consider their future careers by seeking direction through senior residents’ and professors’ positive or negative influences. Through duty transfer and reporting with senior doctors, interns developed confidence working in a hospital setting and experienced psychological support. As role models, professors set the framework for hospital work and proper behaviour, attitude, and values.

*At first, I thought I had to know everything to make a prescription. I came to understand that there was no big problem in administering medicine based on previous experience. As an intern attending physician, I wasn’t so scared about giving medication prescriptions… (Participant 4)*

Participant 4, who was conflicted between ophthalmology and internal medicine, decided on internal medicine because of a respected professor’s influence. *‘I just want to be like the professor. It’s not that the professor did something great for me, but I want to be just like what the professor’s shown me’ (Participant 4).*

#### Clinical department situational awareness

Career identity is in part a reconstruction of past experiences of complex situations, providing a basis for a critical view of professional values [6]. The interns used their challenging experiences in clinical departments when considering their future careers.

*I considered psychiatry, but contrary to my understanding, often it was just medication. There wasn’t even a variety of medication, so it was different from what I imagined…As for paediatrics, I was dispatched there, and I cared for many feverish and sick babies. I had a hard time, and it was difficult dealing with the parents. (Participant 5)*

*Can I hold on for a long time? …There were many departments that didn’t have competition as I had expected and departments where I didn’t compete and didn’t stay for a year. There are many people who switched last minute because of this. (Participant 11)*

Participants also discussed experiences related to gender discrimination, a problem evidenced by recent statistics of gender discrimination in the selection process, attributed to the male-oriented social environment [19].

*In fact, there is implicit preference for men in all departments. The same goes for orthopaedics. If three applicants are being chosen from four men and one woman, I think that they will probably pick three men and exclude me… (Participant 6)*

#### Relationship experience

Career identity becomes a semantic structure that seeks to transform into action through external dialogue with others [8, 20], leading to self-directed career management and development and resulting in active career behaviours [6, 21]. The category ‘preliminary approval of decision by the department’ relates to behaviours involving interns demonstrating their wish to be selected for a particular department. ‘Experience of the relationship with patients’ is considered an expression of self-directed career management of an individual seeking growth and development while contemplating their future role as a doctor.

*In rehabilitation, I did a lot of sore dressing. I didn’t just do the dressing in silence but asked [the patients] about their condition, talked, and shared things we were having difficulties with. It was pleasant to have such conversations, and I got closer to the patients. (Participant 5)*

### C. System domain

The system domain comprises categories related to the ‘experience of institutional limitation’ that participants faced in the Korean internship system. Several participants mentioned that internships improved their understanding and perceptions of the departments, but that this also resulted in interns rejecting specialties lacking senior residents or experience with interns. Consequently, there was dissatisfaction with the overall internship policy, and it was considered a result of the structural problem of unreasonably low payments from the medical insurance system in Korea (Table 3).

*I can’t help but avoid a certain department more if I see too many bad things. But you can’t help but dislike a department you are avoiding. It shows…I care about my future. (Participant 7)*

*There is a month-long cycle, so there are too many departments I can’t get to. (Participant 9)*

**Table 3:** System domain

Domain	Category/Subcategory	Frequency
System domain	1) Experience of institutional limitation	
	a. Not choosing a specialty that lacked senior residents	Variable (4)
	b. Not choosing a specialty programme that had no internship experience	Variable (3)
	c. Dissatisfaction with the overall internship policy because of the structural problem of medical fees in Korea	Typical (6)

## Discussion

This study examines how complex factors and interactions, experienced by interns in Korea, affect their career identity formation [6]; career identity provides a foundation for professional values while promoting personal goals and aspirations, which is important for future doctors.

Participants in this study recognised hospital internships as venues for developing career competencies by adapting to the new environments of hospitals; interns developed awareness of their careers while continuing the learning process from the personal cognitive domain perspective. While recognising the need for this training process, many interns had complaints about the system and developed their own strategies to overcome its limitations.

Korea has no government support for intern/resident education. Recently, a law to improve resident training environments and resident status was enacted, along with policies regarding national responsibilities, training rules for patient safety, and fostering excellent medical personnel. Considering this trend, national support should be provided for developing general competencies as well, including professional identity training.

As demonstrated by previous studies, mentoring is a career tool that helps mentees acquire skills necessary in their roles, while its psychosocial function is to encourage appropriate behaviours, attitudes, and values [22]. It also has a role modelling function, in which mentees emulate the mentor [23]. Participants in this study experienced these functions through their internships. Senior residents are mainly responsible for career-related functions, while professors have psychosocial and role model functions.

This study found that acquiring work-related knowledge, learning through challenging work, and learning through interaction with others, essential to career identity development [24], are limited by individual efforts alone. From a social interaction domain perspective, interns were able to make good use of the interactions with senior residents or specialty professors. However, the system of internship training itself was considered a negative experience of the reality of the specialty and sometimes had a discouraging influence on participants, such as participants changing careers in consideration of competitive pay rate and gender discrimination. This trend is being manifested in Korea by doctors avoiding specialties such as general surgery, thoracic surgery, obstetrics, and gynaecology. This phenomenon has various causes such as low medical insurance payment system, tough working environments and extremely difficult training, and uncertainty of career options after training [25].

In Korea, popular departments receive numerous applications for specialty selection, whereas the less popular departments have difficulty recruiting residents. These issues have led to the practice of ‘preliminary approval of decision by the department’. Within this practice, the relevant professors interview residency applicants or check their reputation and school grades prior to the official open recruitment. They then notify candidates who will be rejected so that they can apply to other departments. This process is often implemented in popular departments and is a way to eliminate competition; thus, candidates’ chances are usually set to 1:1 when selecting specialties. The problems of ‘preliminary approval’ is that objective data, such as school grades, internship grades, and paper tests; are excluded from the evaluation because of the numerous inquiries and prejudices of professors with substantial influence in the department. Furthermore, there is a possibility that the applicant’s connections work in their favour, or that they experience discriminatory factors such as women’s exclusion [26]. To solve the ‘preliminary approval of decision by the department’ derived from this research, it is necessary to improve the specialty selection process.

Career identity can produce useful and valuable results through reflection on experiences and leads to continuous growth through interaction with the environment [8]. It is formed through individuals’ interaction with their environments [27]. The present study confirmed that career identity was established by cyclically interacting with others including senior residents, professors, and other school staff.

In Korea, doctors must choose a specialty through an internship within a period of 1 year. The present study confirmed that there are system constraints limiting interns’ ability to fully grasp various experiences and clinical departments’ characteristics. As a result, interns refrain from selecting specialty departments that lack senior residents or previous experience providing internship training. The participants had many complaints regarding poor national support for internships and the structural problem of medical fees.

The internship programme in Korea serves a role similar to that of the resident matching programme in the US, but interns are unable to work in all departments within a year, and, unlike in most OECD countries, a designated training hospital in Korea is fully responsible for the interns, including the total cost of specialty selection and education. Thus, Korean interns/residents, who have dual positions as trainees and employees, are not provided individual career management and various other support due to high costs.

The study found that 7 out of 11 participants changed their specialty after their internships. This is significant as satisfaction with the selected specialty has been shown to affect several other areas, including satisfaction as a doctor, patient satisfaction, and quality of medical care [2]. In particular, transparency in the resident selection process should be improved. It was found that during internships, the closed nature of the selection process was a barrier for interns’ application to specific departments, where positions are ultimately filled by applicants who have previously expressed their will to apply. In addition, gender discrimination occurs in some departments. Securing transparency in the resident selection process requires developing non-discrimination guidelines for resident selection.

Steffy and Jones [28] suggest that people with clear career goals have more specific career plans and are more immersed in their careers. Furthermore, such individuals are more likely to succeed in career management [29].

The detailed topics derived from the participants’ interviews included some issues that had been raised in some research reports or discussions regarding finding ways to improve the internship system. Considering their importance to interns, the government ought to look for solutions to these problems, rather than leave them to the training hospital.

This study provides a base for developing institutional and policy support for students during an uncertain time when they must choose a specialty before finishing a one-year training course. We examined internal and external factors influencing the important period of career identity formation that determines the future career direction for intern graduates who have recently decided on a specialty. Regretfully, information about the career identity formation of residents was not collected. Residency is also a training course before becoming a specialist, and how the experience before and after deciding on a specialty can affect residents’ career identity formation could have many implications for individuals and their environments and should be the subject of a longitudinal study. Additionally, guidance specialists and senior residents have a significant impact on career identity. We will be able to determine various implications through further studies, and we look forward to future studies examining the formation of career identity of others in the medical field and in other professional fields.

## Conclusions

The internship programme provides information about clinical departments to applicants seeking residency; it is an important period during which career identity is formed. This study helps provide an in-depth understanding of interns and a base for developing institutional and policy support for students during an uncertain time when specialties should be selected.

## Data Availability

The datasets used during the study are available from the corresponding author on reasonable request.

## Abbreviations

CQR: Consensual qualitative research
OECD: Organization for economic cooperation and development
